# Pearls and pitfalls in contemporary management of marginal velopharyngeal inadequacy among children with cleft palate

**DOI:** 10.3389/fped.2023.1187224

**Published:** 2023-08-07

**Authors:** Qirong Mao, Jingtao Li, Xing Yin

**Affiliations:** ^1^ Department of Oral and Maxillofacial Surgery, State Key Laboratory of Oral Diseases, National Clinical Research Center for Oral Diseases, West China Hospital of Stomatology, Sichuan University, Chengdu, China; ^2^ Department of Orthodontics, State Key Laboratory of Oral Diseases, National Clinical Research Center for Oral Diseases, West China Hospital of Stomatology, Sichuan University, Chengdu, China

**Keywords:** cleft palate, velopharyngeal dysfunction, speech therapy, pharyngoplasty, palate lengthening

## Abstract

Marginal velopharyngeal inadequacy (MVPI) is a particular status of velopharyngeal closure after cleft palate repair. The physiological and phonological characteristics of patients with MVPI are significantly different from those with typical velopharyngeal insufficiency. The pathological mechanisms and diagnostic criteria of MVPI are still controversial, and there is limited evidence to guide the selection of surgical and non- surgical management options and a lack of recognized standards for treatment protocols. Based on a systematic study of the relevant literatures, this review identifies specific problems that are currently under-recognized in the diagnosis and treatment of MVPI and provides guidelines for further exploration of standardized and reasonable intervention protocols for MVPI.

## 1. Introduction

Congenital cleft palate is one of the most common craniomaxillofacial birth defects in humans and may affect important physiological functions including speech, mastication, swallowing, hearing, and maxillofacial growth and development. Although primary cleft palate repair restores the continuity of soft and hard tissue and physiological anatomy of the palate, a significant percentage of patients still fail to fully close the velopharyngeal port during speech after surgery. The state coined as velopharyngeal inadequacy (VPI) results in varying degrees of speech dysfunction, seriously affects the quality of life and requires further medical intervention (1, 2). More specifically, the speech of patients with VPI is usually characterized by nasal emission, hypernasality and compensatory errors in articulation. And the severity of speech abnormality is associated with the extent of incomplete velopharyngeal closure.

There is no clear-cut distinction between normal and abnormal velopharyngeal closure, and a borderline closure exists between typical VPI and definitive velopharyngeal competent (VPC), which may appear as a mildly incomplete or unstable closure. Since the speech performance and treatment prognosis under the borderline state are significantly different from typical VPI or VPC, it has been classified as a separate diagnostic category as marginal velopharyngeal inadequacy (MVPI), which is widely used to date to evaluate the outcome of cleft palate management. However, there is no unified standard for the diagnosis of MVPI, and different studies not only use drastically different criteria but also differ in defining MVPI as a treatment success or failure, which seriously undermines the cross-sectional comparability of data related to cleft palate treatment outcomes. In addition, intervention options for MVPI are controversial. While the selection of surgery for typical VPI and speech training for VPC with articulation error are indisputable, the structural, physiological, and habitual factors affecting velopharyngeal fuction are often intertwined in MVPI (3). The differences in the willingness to improve speech among patients and the health economic considerations make the choice of surgical and non-surgical treatments for MVPI even more complicated (4–7).

In concern of the controversies in the management of MVPI, we systematically reviewed the research progress related to the pathological mechanism, diagnostic criteria and treatment outcomes of MVPI, so as to clarify the existing research difficulties, misunderstandings and shortcomings and provide reference for further improvement of MVPI treatment protocol.

## 2. Pathogenesis of MVPI

The concept of MVPI was first defined in 1976 as “mild or intermittent incomplete closure” and “a borderline status between complete and incomplete closure” (8). Among patients with MVPI, structural, physiological and habitual factors affecting velopharyngeal closure are intertwined. For example, articulatory errors and mild soft palate elevation deficits may be of reverse causality. It is often difficult to clearly distinguish the various pathological causes of speech intelligibility.

Smith Guyette (1996) suggested that the status of MVPI is where the velopharyngeal closure system demonstrates different degrees of competence in response to speech tasks of varying difficulty. Using sounds of /Pa/ and /Pi/ as a criterion, he observed complete closure of the /Pa/ sound and incomplete closure of the /Pi/ sound in some patients and speculated that there may be antagonistic action of the palatoglossal muscle against the levator veli palatini in /Pi/ sound, resulting in increased resistance to soft palate uplift and incomplete closure (9).

Warren made close observation on the timing of nasal emission and found that the closure phase in patients with MVPI was about 50 ms behind the normal population, resulting in a longer duration of nasal emission and a shorter duration of closure during functional speech. He then suggested that the pathological basis of MVPI may be related to the response timing of the velopharyngeal closure system (10).

Karnell et al. analyzed the pathological characteristics of velopharyngeal closure based on objective nasometer values and observed that some patients showed normal values when completing high-pressure test sentences containing stress consonants and abnormal values when completing low-pressure test sentences containing only vowels and semivowels. On this basis, they proposed the pressure-sensitive theory, suggesting that the velopharyngeal performance of patients with MVPI was influenced by the oronasal pressure associated with the speech task, showing “mixed” nasometer results (3, 11).

Morris hypothesized that MVPI might be categorized into two subtypes: the structural and functional. In the former, complete closure is not possible due to the structural constraints and should be manifested as persistent mild nasal emission, while in the latter, the velopharyngeal mechanism meets the requirements for closure but is affected by poor articulatory habits and demonstrates incomplete closure during difficult speech tasks, which should be manifested as intermittent nasal emission (12). This subtype classification hypothesis, however, has not yet been supported by adequate research data.

To date, there is no uniform understanding of the pathological mechanisms underlying the development of MVPI. Different studies often focused on only one aspect of speech performance. The lack of an exact pathological mechanism also leads to controversies regarding the diagnostic criteria for MVPI.

## 3. Diagnostic criteria of MVPI

Early in the introduction of the MVPI concept, there was debate on whether it should be classified as a transitional state on the continuous spectrum of VPI or as a third diagnostic classification distinct from typical VPI and VPC. With increasing evidence suggesting distinct performance in hypernasality and speech among patients with MVPI from those with VPI and VPC, and the clinical significance of MVPI diagnosis to treatment options and prognosis, MVPI became recognized as an independent diagnostic category and widely used in the evaluation of cleft palate treatment outcomes (3, 13). The diagnostic criterion of MVPI is yet to be unified. Accordingly, the identification between MVPI and mild VPI becomes critical to their clinical management.

The tools for velopharyngeal function evaluation include subjective evaluation, endoscopy, radiography, nasometer, and oronasal pressure test. Early studies mostly used a single examination for diagnosis, such as classifying patients with a velopharyngeal gap less than 2 mm on lateral radiographs as MVPI (14). Although diagnostic methods based on a single examination generally showed good internal consistency, the agreement between the results of different examination methods is often low. For example, more than half of the patients with a velopharyngeal gap less than 2 mm turned out to be definitive VPI or VPC (15).

Laine et al. made definitive diagnosis on velopharyngeal function basing solely on the ventilation port size deduced from nasometer values: a port less than 0.05 cm^2^ was diagnosed as VPC, 0.05–0.09 cm^2^ as marginal complete velopharyngeal closure, 0.10–0.19 cm^2^ as marginal incomplete velopharyngeal closure, and more than 0.20 cm^2^ as VPI (16). Warren et al. based their diagnosis algorithm on the severity of the hypernasality on a scale of 1–4, with VPC below 1.676, marginal complete velopharyngeal closure from 1.677 to 2.368, marginal incomplete velopharyngeal closure from 2.369 to 2.5, and VPI above 3.273 (17). Morris relied entirely on subjective evaluation for diagnosis, using a speech scale to obtain an overall score for nasal emission, resonance, and articulation. A score of 3–4 on the scale (1 for normal and 7 for severe abnormalities) was used as diagnostic criterion for MVPI (13). In addition, Morris (18) proposed to divide MVPI into two subtypes: “almost but not quite” (ABNQ) and “sometimes but not always” (SBNA). In the former, the velopharyngeal closure is always incomplete and there is a consistent mild nasal emission during pronunciation, whereas in the latter, complete closure can be achieved occasionally but not consistently (19). However, mild and intermittent incomplete closure could not simply equate to structural and functional causes (20). Although this subtype classification has potential value to intervention selection, its existence is not supported by the currently available data.

Concerning the limitations of a single examination, it is now believed that the diagnosis of MVPI should combine subjective and objective findings, and a three-dimensional and dynamic endoscopic evaluation of velopharyngeal port is generally recommended (21, 22). There are still drastic differences in the diagnostic criteria of MVPI reported in the literature, and more studies involving MVPI did not even clearly describe their diagnostic criteria (23–25). Moreover, there is no agreement on whether to classify MVPI as a successful or unsuccessful outcome for cleft palate treatment, which seriously affects the comparability among studies (26–28). A definite and explicit diagnosis criterion is prerequisite to intervention selection, and inconsistency in diagnosis inevitably leads to different management protocols for MVPI in different institutions.

## 4. Disagreements on the management philosophy of MVPI

The treatment options for typical VPI and VPC are relatively clear, with the former requiring further surgery to restore the velopharyngeal mechanism and the latter relying on speech training to correct habitual errors (29). In contrast, it is often difficult to draw a definitive line between structural velopharyngeal abnormalities and articulation abnormalities in patients with MVPI (30). Mild closure inadequacy may force compensatory articulation, and normal articulation may be restored after surgery without speech training, while abnormal articulatory habits may affect velopharyngeal closure and velopharyngeal closure insufficiency may disappear after correction of articulation (31, 32). In addition, MVPI status may be less stable during the follow-up as compared to VPI and VPC, further complicating the clinical decision-making (33, 34).

In an idealized medical setting, it seems reasonable to firstly prescribe speech training for all patients with MVPI and subsequently schedule surgery according to the training outcome. Speech training can determine whether articulation error is the initiating factor in causing speech problems and does not cause structural changes at the velopharyngeal port (4). In a realistic medical setting, however, the time and financial costs associated with speech training are not affordable for all families, especially in remote regions where speech therapy is not yet available (35–37).

The other extreme of the MVPI management is to prescribe indiscriminate surgery. Some scholars believed that patients with MVPI generally yielded poor outcomes to speech training and prefer to perform surgical intervention first (5, 12, 38). Some studies found that re-palatoplasty improves the outcome of speech training among patients with MVPI, probably because surgery makes it easier for them to achieve complete closure and master the correct articulation techniques with resonance and emission eliminated (5, 6). However, this strategy is at risk of overtreatment and complications to patients who may be potentially cured by speech training alone.

Thus, assessment of the sensitivity of MVPI patients to speech training seems to be an important prerequisite for the development of an accurate treatment plan. This philosophy was reflected in the ABNQ and SBNA category proposed by Morris, who suggested that the former is difficult to achieve further improvement through training and therefore suitable for surgical treatment, while the latter has more potential in velopharyngeal mechanism that could be activated by speech training (12, 18, 19). However, there are no reliable data to support the accuracy and reliability of Morris’ subtype classification to guide clinical treatment.

In addition, the willingness of patients with MVPI patients is polarized. Some patients get along well with mild speech abnormalities in daily life and are not willing to undergo surgery, while some patients believe that their speech is close to completely normal and hold high expectations for the last step of treatment. Patients’ attitude and expectation also play an important role in making treatment decisions (39, 40).

In view of the above-mentioned issues, the literatures have not yet formed a well-recognized standard treatment standard for MVPI. The identification of MVPI from mild VPI and the decision on corresponding management protocol are still highly subjective. Physicians are generally suggested to develop individualized treatment plans depending on their experience, which obviously lacks practical guidance. The lack of evidence for the clinical management of MVPI is the main reason for the absent of standardized protocols.

## 5. Clinical intervention options for MVPI

### 5.1. Speech therapy

Patients with MVPI are of their own characteristics in speech, usually not demonstrating all the typical problems of VPI in terms of hypernasality, emission and articulatory error, and their speech performance may vary in coping with different speech tasks. For example, Karnell et al. concluded that low-pressure test sentences were more likely to induce hypernasality in patients with MVPI. Speech spectrum analysis likewise suggested that pressure consonants were most likely to be abnormal in patients with MVPI (21, 41). It has been shown that about 80% of patients with MVPI demonstrated phonological problems (13, 21) and their common articulation errors included vowel omission and post-phonological articulation. Therefore, individualized speech therapy should be design for each patient. For example, the treatment of vowel omission should focus on target sound elicitation, and the treatment of post-phonological articulation should focus on moving the articulation position forward.

In addition, Continuous Positive Airway Pressure (CPAP) has also been used to assist speech training. Theoretically, CPAP provides a resistance training scenario for velopharyngeal closure and helps to strengthen the relevant muscles (42). It has been shown that CPAP could be potentially effective in improving the efficiency of speech training and reducing the difficulty of eliciting target sounds (36). This instrument, however, has not been widely employed among cleft centers. Accordingly, publications concerning the effectiveness of CPAP in facilitating cleft speech training are highly limited. Further studies with decent patient volume and well-controlled design are required.

Although some small-sample studies reported good speech training outcome in all included patients with MVPI (43–45), cases with poor outcomes would be more valuable from the perspective of clinical guidance. For example, it was found that MVPI patients who responded poorly to speech therapy and required surgery intervention tended to have articulation errors in low-pressure speech-length sentences (21), which was consistent with the pressure-sensitivity theory. Analysis of the prognostic factors of speech training has significant medical-economic value and can help in the choice between surgical and non-surgical strategies in the treatment of MVPI. However, such relevant studies are currently scarce and the findings of individual studies need further validation.

### 5.2. Surgical intervention

Surgical interventions become necessary when speech therapy fails to correct the closure insufficiency or when the patient is unwilling to take speech training. The surgical options for VPI after cleft palate repair include palatal lengthening and pharyngoplasty (46). Given the potential risk of nasal airway obstruction after pharyngoplasty and the high velopharyngeal closure rate in patients with MVPI, palatal lengthening generally preferred.

The most widely used technique for MVPI in the literature is the reverse double-Z approach proposed by Furlow and its modifications (38, 47, 48). This procedure effectively lengthens the soft palate and tightens the musculature posteriorly to improve velopharyngeal closure. Sommerlad's technique has also been used in the treatment of MVPI. This technique is more radical in soft palate muscle reconstruction than the double-Z technique (49). Mann et al. proposed to transfer the buccal mucomuscular flap to better lengthen the soft palate and has also been successfully used in the treatment of VPI after cleft palate repair (50). In addition, posterior pharyngeal wall augmentation by filling of various materials has been reported to be successful in managing VPI but is currently not the first-line option for MVPI treatment in most cleft centers due to the resorption, translocation of fillers as well as related biosafety risks (51–53).

Yamaguchi et al. reported that 78.57% of 42 patients with MVPI had complete velopharyngeal closure after Furlow procedure as evidenced by endoscopic examination (29). Hsu et al. reported satisfactory velopharyngeal function in 13 patients who underwent the Furlow procedure, with 69% of them achieving normal resonance and 80% of them having resolution of nasal emission (26).

Otherwise, studies are highly limited on the outcome and prognosis-related factors of MVPI management. Notably, the inconsistency of diagnostic criteria for MVPI seriously affects the comparability across studies. Clinical studies on MVPI treatment needs to incorporate a comprehensive panel of measurements including time-economic burden, surgical risk, and speech benefit.

The limitations of this review must be noted. The largest limitation is the controversial diagnostic standards employed in different studies, which significantly debilitated the inter-study comparability. Most studies simply provided no or vague description on their MVPI diagnostic methods. Second, the sample sizes are generally small in studies concerning MVPI outcomes. Third, no study comparing different management protocol or surgical techniques has been reported, forcing the authors to speculate the most appropriate suggestion on several cricical decision-making points in MVPI management. These limitations exist throughout the discussion in this manuscript.

## 6. Conclusions

The diagnostic criteria and treatment protocols of MVPI are currently controversial. Studies on variables that may influence the outcome of speech assessment will help to clarify the mechanisms underlying the possible discrepancy between speech performance and velopharyngeal function. Clinicians should base their MVPI management protocol on the most repeatable and reliable diagnostic criteria in their institutions. Speech therapy for either diagnostic or treatment purpose is suggested prior to surgery. Palate lengthening is preferred over pharyngoplasty in the surgical management of MVPI. Explorations on the prognostic factors will provide further evidence-based guides for MVPI management.
